# Development of drug-inducible CRISPR-Cas9 systems for large-scale functional screening

**DOI:** 10.1186/s12864-019-5601-9

**Published:** 2019-03-19

**Authors:** Ning Sun, Sakina Petiwala, Rui Wang, Charles Lu, Mufeng Hu, Sujana Ghosh, Yan Hao, Christopher P. Miller, Namjin Chung

**Affiliations:** 1 AbbVie Inc., 1 North Waukegan Rd., North Chicago, IL 60064 USA; 2 AbbVie Cambridge Research Center, 200 Sydney St, Cambridge, MA 02139 USA

**Keywords:** CRISPR, Gene editing, Functional genomics

## Abstract

**Background:**

Large-scale genetic screening using CRISPR-Cas9 technology has emerged as a powerful approach to uncover and validate gene functions. The ability to control the timing of genetic perturbation during CRISPR screens will facilitate precise dissection of dynamic and complex biological processes. Here, we report the optimization of a drug-inducible CRISPR-Cas9 system that allows high-throughput gene interrogation with a temporal control.

**Results:**

We designed multiple drug-inducible sgRNA expression vectors and measured their activities using an *EGFP* gene disruption assay in 11 human and mouse cell lines. The optimal design allows for a tight and inducible control of gene knockout in vitro, and in vivo during a seven-week-long experiment following hematopoietic reconstitution in mice. We next performed parallel genome-wide loss-of-function screens using the inducible and constitutive CRISPR-Cas9 systems. In proliferation-based dropout screens, these two approaches have similar performance in discriminating essential and nonessential genes. In a more challenging phenotypic assay that requires cytokine stimulation and cell staining, we observed similar sensitivity of the constitutive and drug-induced screening approaches in detecting known hits. Importantly, we demonstrate minimal leakiness of our inducible CRISPR screening platforms in the absence of chemical inducers in large-scale settings.

**Conclusions:**

In this study, we have developed a drug-inducible CRISPR-Cas9 system that shows high cleavage efficiency upon induction but low background activity. Using this system, we have achieved inducible gene disruption in a wide range of cell types both in vitro and in vivo. For the first time, we present a systematic side-by-side comparison of constitutive and drug-inducible CRISPR-Cas9 platforms in large-scale functional screens. We demonstrate the tightness and efficiency of our drug-inducible CRISPR-Cas9 system in genome-wide pooled screening. Our design increases the versatility of CRISPR-based genetic screening and represents a significant upgrade on existing functional genomics toolbox.

**Electronic supplementary material:**

The online version of this article (10.1186/s12864-019-5601-9) contains supplementary material, which is available to authorized users.

## Background

The clustered regularly interspaced short palindromic repeats (CRISPR)/CRISPR-associated protein 9 (Cas9) is a transformational toolset for mammalian genome engineering [1–3]. Guided by a single guide RNA (sgRNA) that recognizes its target DNA sequence through complementary base paring, Cas9 protein generates a double-strand break in the genome in a highly specific and efficient manner. The subsequent repair of the DNA cleavage by error prone non-homologous end joining often leads to small insertions or deletions at the target site, resulting in gene disruption. Given its efficiency and scalability, the CRISPR-Cas9 system has been rapidly adopted for genome-wide loss-of-function genetic screens in order to dissect gene functions in various biological processes, including proliferation, drug resistance, viral infection, metabolism and metastasis [4–8]. However, most screens rely on the constitutive endonuclease activity of CRISPR-Cas9 where Cas9 and its sgRNA are constantly co-expressed. This can limit certain applications in which genome editing processes need to be precisely controlled temporally. An inducible CRISPR-Cas9 system allowing temporal control of its genome editing activity would broaden its versatility and enhance its ability as a research tool for functional genomics.

Previously, multiple strategies have been developed to control the timing of CRISPR-Cas9 activity through an external signal such as a small molecule or light irradiation [9–29]. It has been shown that conditional gene editing can be achieved by regulating the expression, stability and nuclease activity of Cas9 or by modulating the availability and conformation of the sgRNA. However, most of the reported systems are limited by design for use in a low-throughput format and their performance in large-scale functional screening has not been evaluated. Here, we developed a chemically-regulated sgRNA expression platform that enables controllable genetic manipulations in mammalian cells on a genome-wide scale. We compared *Tet* and *Lac* operator-repressor gene regulatory systems to control sgRNA transcription. Upon rigorous evaluation of multiple designs, we identified optimized systems that deliver highly potent gene editing upon chemical induction with negligible background activity. We have demonstrated the efficacy of our systems in 12 human and mouse cell lines in vitro. Additionally, we benchmarked our systems against reported chemical-inducible methods [18, 24] and demonstrated their superiority. We also tested our construct in primary hematopoietic progenitor cells in an in vivo reconstitution mouse model [30]. Following the seven-week hematopoietic reconstitution, our system remained tight in non-induced conditions while allowed efficient in vivo gene interrogation in various cell types that developed from this transduced progenitor pool after induction. Finally, we systematically characterized our constructs in a proliferation-based negative-selection screen to identify essential genes for sustained cell growth, and in a positive-selection screen based on fluorescence-activated cell sorting (FACS) to isolate programmed death-ligand 1 (PD-L1) regulators. In the absence of induction, we did not detect any evidence of gene editing. In contrast, with induction, we identified the top genes predicted to be impacted by gene editing in our genome-wide screening setting. To our knowledge, this is the first time anyone has demonstrated the tightness and efficiency of an inducible CRISPR-Cas9 genome editing system in genome-wide screening. Our drug-induction approach expands the CRISPR toolbox and has great potential to accelerate functional genomics for uncovering novel cellular mechanisms and identifying new drug targets in a temporal desired setting.

## Results

### Initial design and testing of multiple drug-inducible CRISPR-Cas9 systems

**Fig. 1:**
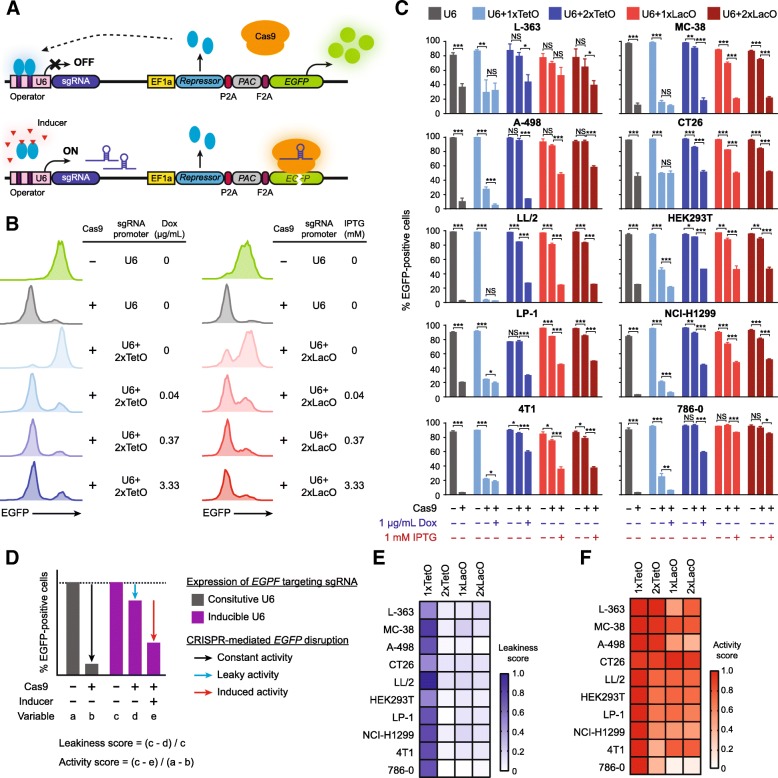
Design and evaluation of drug-inducible sgRNA expression vectors**.** (**a**) Schematic for drug-inducible sgRNA expression vectors. Cas9 is constitutively expressed in the cells. *EGFP* reporter gene is used for the quantification of genome editing activity. *PAC* encodes puromycin N-acetyltransferase. (**b**) Representative flow cytometry histograms showing dose-dependent inducible *EGFP* knockout in MC-38 for *tet*- (left) and *lac*- (right) systems. (**c**) Evaluation of background activity and drug inducible gene knockout efficiency of the inducible sgRNA expression vectors in multiple cell lines. Data represent mean ± SD (*n* = 3). *P* values were derived from *t* tests: *P < 0.05; **P < 0.01; ***P < 0.001; NS, nonsignificant. (**d**) Calculation of leakiness score and activity score. (**e**) Heat map of leakiness scores. (**f**) Heat map of activity scores

We sought to regulate the DNA cleavage activity of the CRISPR-Cas9 system by modulating the availability of sgRNA because Cas9 must associate with sgRNAs to exert its function. The Cas9 gene under the control of the EF1a promoter was first introduced into target cells through lentiviral transduction and stable selection. We then engineered a lentiviral vector comprised of a drug-inducible sgRNA cassette and an EF1a promoter driven tetracycline (Tet) repressor (TetR) or lactose (Lac) repressor (LacI) linked via a self-cleaving 2A peptide to the puromycin-selectable marker (Additional file 1 :Figure S1). The sgRNA is driven by a modified U6 promoter, which contains operator sites (TetO or LacO) that allow for suppression of sgRNA transcription in the presence of TetR or LacI, respectively. Addition of chemical inducers, doxycycline (DOX) for the Tet-system and isopropyl β-D-1-thiogalactopyranoside (IPTG) for the Lac-system, causes the repressors to dissociate from their respective operators, allowing for efficient sgRNA transcription (Fig. 1a). To fine-tune the balance between background sgRNA expression and drug-induced gene editing, we inserted one or two copies of their respective operators into the U6 promoter for the Tet- and Lac-inducible systems (termed 1xTetO, 2xTetO, 1xLacO and 2xLacO, respectively, Additional file 1 :Figure S1&S2). We employed a reporter system based on enhanced green fluorescent protein (EGFP) expression to evaluate gene editing efficiency. Briefly, Cas9-expressing cells are infected with a lentivirus carrying both the *EGFP* gene and an sgRNA targeting *EGFP*. CRISPR-mediated *EGFP* cleavage and subsequent NHEJ will lead to loss of EGFP expression (Fig. 1a). As a proof of principle, we transduced both parental and Cas9 stable MC-38 cells with this reporter and observed significant reduction of EGFP signal in the cells expressing both active Cas9 and *EGFP*-targeting sgRNA as compared to those lacking Cas9 (Fig. 1b). In order to evaluate various designs for conditional drug-induced gene editing, we constructed multiple EGFP reporter plasmids delivering the *EGFP*-specific sgRNA under the control of modified U6 promoters consisting of 1xTetO, 2xTetO, 1xLacO or 2xLacO, respectively. After transduction and puromycin selection, the Cas9 stable MC-38 cells were treated with various concentrations of inducers for more than five days and then analyzed by flow cytometry. The sgRNA expression was tightly controlled in the 2xTetO system since EGFP expression was only minimally affected in the non-induced state (Fig. 1b&c). In MC-38, gene editing following DOX induction of sgRNA expression was as efficient as that achieved by a constitutive sgRNA expression vector at all tested concentrations of DOX (Additional file 1 :Figure S3). Notably, the vector with only one copy of TetO in the U6 promoter showed high DNA modification rates both in the presence and absence of DOX, suggesting insufficient transcription inhibition of sgRNA by TetR in the 1xTetO design (Fig. 1c and Additional file 1 :Figure S3). We observed strong dose-dependent control of gene editing activity for both 1xLacO and 2xLacO systems. Increasing levels of *EGFP*-disrupting efficiency were observed upon treatment at increasing concentrations of IPTG and reached a maximum at 1 mM (Fig. 1b&c and Additional file 1 :Figure S4).

To explore the generalizability of our drug-inducible systems, we tested them in a broad spectrum of murine and human cell lines from various tissues. Consistent with the results obtained in MC-38, the 1xTetO system exhibited high background activity in the absence of DOX, even though the efficiencies of DOX-induced gene knockout reached to the same level as in the constitutive setting (Fig. 1c and Additional file 1 :Figure S3). To the contrary, when an additional TetO was added to the U6 promoter in the 2xTetO design, there was minimal and often insignificant background activity in the absence of DOX and high cleavage efficiency upon DOX treatment (Fig. 1c and Additional file 1 :Figure S3). The 1xLacO and 2xLacO designs exhibited subtle off-state activity and efficient gene knockout by the addition of IPTG (Fig. 1c). In addition, our dosage-dependent experiment suggests that maximum activity can be achieved at 1 mM IPTG in all cell lines examined (Additional file 1 :Figure S4). For each system, we defined its leakiness score as the ratio of the loss of EGFP signal in the absence of inducers as compared to the EGFP level in the cells without Cas9 expression. We also calculated the activity score by dividing frequency of *EGFP* disruption in the presence of inducers by that achievable with constitutive sgRNA expression system (Fig. 1d). Whereas the 1xTetO design had high leakiness score and activity score, 2xTetO system showed minimal leakiness (0–14%) and high activity score (39–99%) across all the tested cell lines (Fig. 1e&f and Additional file 1 :Table S1). Compared to the 1xLacO system, which demonstrated 0–21% leakiness and retained 10–97% of the constitutive activity in the presence of IPTG, the 2xLacO system exhibited lower leakiness (0–17%) while maintaining similar activity upon IPTG addition (Fig. 1e&f and Additional file 1 :Table S1). Collectively, the 2xTetO and 2xLacO designs demonstrated favorable performances in regard to their low leakiness and high efficiency, which were selected for further characterization.

### Further characterization and benchmarking against other chemical-inducible designs

**Fig. 2:**
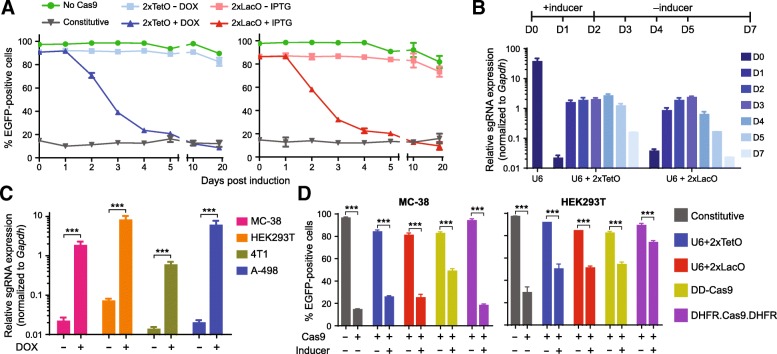
Further characterization of drug-inducible CRISPR platforms and benchmarking against literature designs. (**a**) Time-course of *EGFP* disruption activity after drug treatment. MC-38 cells with stable Cas9 expression were transduced with constitutive (grey lines), DOX-inducible (light and dark blue lines) and IPTG-inducible (light and dark red lines) sgRNA expression vectors and selected with puromycin. Parental MC-38 cells were transduced with an *EGFP* reporter as a control (green lines). 1 μg/mL DOX or 1 mM IPTG was used to induce the sgRNA expression. (**b**) RT-qPCR analysis of sgRNA levels in MC-38 cells. Chemical inducers were applied to cell culture medium on Day 0 and washed out on Day 2. 1 μg/mL DOX or 1 mM IPTG was used to induce the sgRNA expression. (**c**) RT-qPCR analysis of sgRNA levels with or without DOX treatment. 1 μg/mL DOX was used to induce the sgRNA expression. Data represent mean ± SD (*n* = 3). *P* values were derived from *t* tests: ***P < 0.001. (**d**) Comparison of background activity and inducible efficiency with existing (DD-Cas9 and DFHR.Cas9-DHFR) designs using the *EGFP* disruption assay. Induction conditions: 1 μg/mL DOX for 2xTetO system, 1 mM IPTG for 2xLacO system, 1 μM Shield-1 for DD-Cas9, and 10 μM TMP for DHFR.Cas9.DFHR. Data represent mean ± SD (*n* = 3). *P* values were derived from *t* tests: ***P < 0.001

To understand the temporal kinetics of our inducible knockout systems, we carried out a time-course analysis of *EGFP* disruption in MC-38 cells. Significant decrease in EGFP expression began to be observed 48 h after treatment with inducers. Furthermore, the editing activities continued to increase with longer treatment durations and reached the same level as those in the constitutive setting 10 days after induction (Fig. 2a). Notably, the background activities in the absence of inducers did not increase with continuous culture for up to 20 days (Fig. 2a), suggesting the background sgRNA expression in our 2xTetO and 2xLacO systems can be tightly controlled in long-term experiments. We also performed quantitative reverse transcription PCR (RT-qPCR) to measure the sgRNA levels from 0 to 48 h following inducer application. We observed minimal sgRNA expression prior to and strong induction (85-fold for 2xTetO and 50-fold for 2xLacO, respectively) after inducer treatment for 48 h (Fig. 2b). In addition, we observed significant decrease of the sgRNA expression upon removal of small molecule inducers. The efficient reversibility of our inducible sgRNA vectors allows for limiting Cas9 nuclease activity within a short temporal window. RT-qPCR analysis confirmed efficient induction of sgRNA expression from 2xTetO cassette upon DOX treatment in multiple murine and human cell lines (Fig. 2c). However, the background sgRNA expression does not correlate with leaky *EGFP* disruption in the absence of DOX (Additional file 1 :Figure S5A). In addition, induced sgRNA expression levels do not correlate with *EGFP* disruption activities by the addition of DOX (Additional file 1 :Figure S5B). An important goal for future studies will be to identify the genomic or epigenetic factors that might affect the tightness and activity of our inducible sgRNA expression vectors in order to further improve their performances in distinct cell types.

Since multiple designs of drug inducible CRISPR-Cas9 systems have been reported previously, we sought to benchmark our DOX- and IPTG-inducible systems against these published designs. We selected a DD-Cas9 design in which Cas9 is linked to an FKBP12-derived derived destabilizing domain so that Cas9 availability can be regulated by a FKBP12 synthetic ligand, Shield-1 [24]. In another design, a structurally unstable protein domain derived from *Escherichia coli* dihydrofolate reductase (DHFR) is fused to both N and C termini of Cas9 (DHFR.Cas9.DHFR), so that the addition of trimethoprim (TMP), a DHFR-stabilizing small molecule, provides inducible control of Cas9 nuclease activity [18]. We cloned both inducible Cas9 derivatives into the same lentiviral vector that we used for the *EGFP* disruption assay and generated MC-38 and HEK293T cell lines that stably express them. Then we performed the *EGFP* disruption assay to compare our systems with these published designs in a carefully controlled manner under the same experiment conditions. Consistent with previous results, we observed minimal background signal and high activity upon inducer application using our DOX- and IPTG-inducible sgRNA expression systems. By comparison, DD-Cas9 design exhibited higher off-state signal in HEK293T and less potent induction in MC-38. The DHFR.Cas9.DHFR displayed insignificant background activity in both MC-38 and HEK293T, but its drug induced activity in HEK293T is significantly lower than our designs (Fig. 2d). We did not observe any detectable cytotoxicity in MC-38 or HEK293T caused by different chemical inducers (Additional file 1 :Figure S6). Addition of chemical inducers did not affect EGFP fluorescence signal or canonical function of the CRISPR-Cas9 system when wild type Cas9 and sgRNA were co-expressed constitutively (Additional file 1 :Figure S7). These observations ruled out the possibility that different performances of inducible systems were a consequence of distinct pharmacological properties of chemical inducers. Collectively, these data demonstrated advantageous performance of our DOX- and IPTG-inducible systems over the two existing designs in terms of leakiness and activity. However, it is possible that the performances of the literature designs can be further improved by optimizing the drug concentration and/or incubation time. They also have the advantage of faster dynamics for controlling Cas9 stability than controlling sgRNA expression used in our design.

### In vivo genome editing using drug-inducible sgRNA expression constructs

**Fig. 3:**
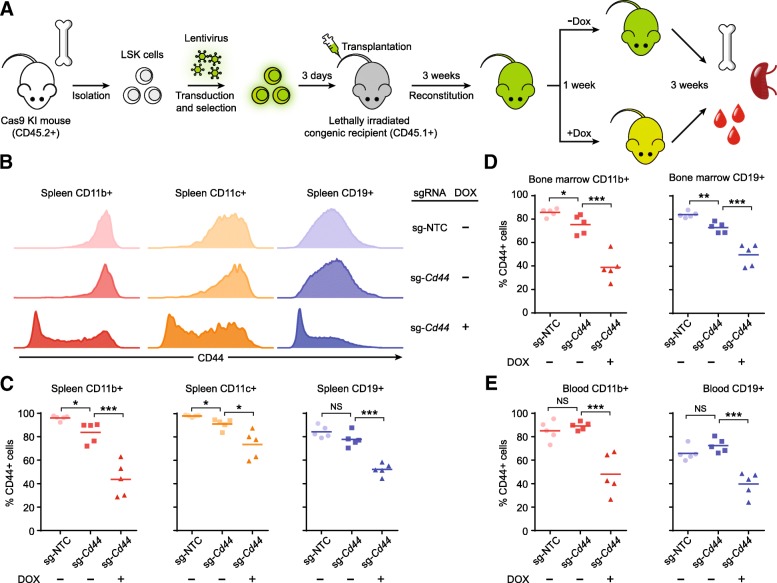
In vivo genome editing using the DOX-inducible sgRNA expression construct. (**a**) Schematic diagram of testing the DOX-inducible sgRNA expression vector in bone marrow chimeric mice. (**b**) Representative flow cytometry histograms showing DOX-inducible *Cd44* knockout in splenic cells. (D) DOX-inducible *Cd44* knockout in CD11b+, CD11c + and CD19+ cells in spleen. (**d**) DOX-inducible *Cd44* knockout in CD11b + and CD19+ cells in bone marrow. (**e**) DOX-inducible *Cd44* knockout in CD11b + and CD19+ cells in peripheral blood. Individual animals and mean (**c**-**e**) are shown with five mice per group. *P* values were derived from *t* tests: *P < 0.05; **P < 0.01; ***P < 0.001; NS, nonsignificant

After demonstrating the efficiency of our conditional gene knockout platforms in cell culture, we sought to explore the applicability of our sgRNA expression system in primary cells in vivo. As a proof of concept, we used our DOX-inducible sgRNA expression construct to temporally control CD44 expression in a hematopoietic reconstitution mouse model. Briefly, we isolated lineage^−^/Sca-1^+^/c-kit^+^ (LSK) cells, which contain hematopoietic stem cells, from the bone marrow of Cas9-expressing transgenic mice (CD45.2^+^) [31]. Next, we transduced those cells with a lentiviral vector expressing the violet-excited GFP (vexGFP) gene under the control of the EF1a promoter and a DOX-inducible sgRNA targeting the murine *Cd44* gene. The transduced cells were then transplanted into lethally irradiated C57BL congenic recipient mice (CD45.1^+^) to generate bone marrow chimeras. Three weeks after transplantation, the mice with their hematopoietic system reconstituted were separated into two groups and fed with regular or DOX-containing food for a week. Three additional weeks following DOX treatment, distinct immune cells from bone marrow, spleen and blood were subjected to flow cytometry analysis (Fig. 3a). We used CD45-based congenic markers to distinguish transplanted cells (CD45.2+) from recipient cells (CD45.1+) and vexGFP to track the cells carrying the inducible sgRNA expression cassette. We demonstrated successful hematopoietic reconstitution, indicated by a high percentage of CD45.2+ cells in all tested organs (Additional file 1 :Figure S8). We also showed that DOX treatment did not change the cell composition in major lineages in the reconstituted immune system (Additional file 1 :Figure S9), nor did it affect CD44 expression in the bone marrow chimeric animals expressing a non-targeting control sgRNA (Additional file 1 :Figure S10). However, addition of DOX caused prominent and ubiquitous reduction of CD44 in the hematopoietic-system-reconstituted mice carrying a DOX-inducible *Cd44*-specific sgRNA expression cassette (Fig. 3b-e). Importantly, we observed only minimal CD44 depletion in the non-induced state, indicating that our inducible CRISPR-Cas9 system remains tight in a long-term experiment (seven weeks) in vivo.

### Genome-wide lethality screens using drug-inducible sgRNA expression libraries

**Fig. 4:**
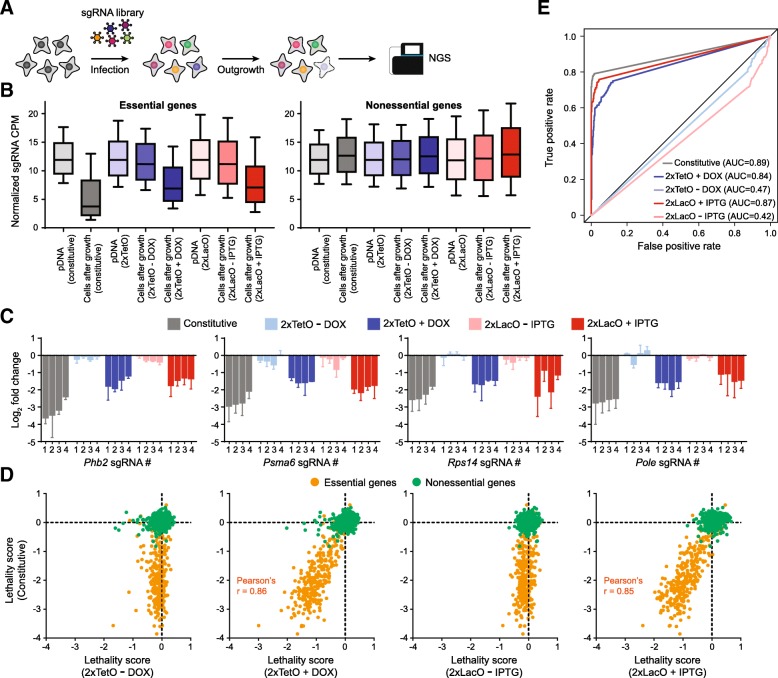
Genome-wide lethality screens using drug-inducible sgRNA expression constructs. (**b**) Schematic overview of proliferation-based negative-selection screening. (**b**) Normalized sgRNA read count distributions for essential and nonessential genes across different samples. CPM, counts per million. (**c**) Fold changes of normalized sgRNA read counts between cells after outgrowth and the original plasmid DNA. (**d**) Scatter plots showing the correlation between the lethality scores of essential (orange) and nonessential (green) genes in the screen using constitutive sgRNA library with those in the screens using inducible sgRNA libraries in the absence or presence of chemical inducers. Pearson correlation coefficient r values for essential genes are shown. (**e**) ROC curves indicating screen performance in identifying essential genes by comparing the library composition between the plasmid library and cells after > 10 population doublings. True positive rates and false positive rates were calculated using a gold-standard set of essential and nonessential genes. The ROC curves are based on the FDR. AUC, area under the curve

In addition to editing individual genes, the CRISPR-Cas9 system has been applied in pooled genetic screening so that the biological functions of a large number of genes can be tested simultaneously. To systematically characterize our drug-inducible CRISPR systems in a large-scale setting, we performed parallel loss-of-function screens in MC-38 cells using a genome-wide pooled sgRNA library (Fig. 4a). We sub-cloned the Brie sgRNA library, comprised of 78,637 sgRNAs targeting 19,674 protein coding mouse genes (~ four sgRNAs per gene) and 1000 non-targeting control sgRNAs [32], into our DOX-inducible and IPTG-inducible constructs with U6 + 2xTetO and U6 + 2xLacO, respectively. The sgRNA libraries were then transduced using lentivirus into MC-38 cells with stable Cas9 expression in three biological replicates. A low multiplicity of infection (MOI = 0.3) was used to minimize lentivirus coinfection. The transduced cells were selected using puromycin and cultured under self-renewal conditions with or without chemical inducers for at least ten population doublings. The sgRNA abundance was then quantitated using next-generation sequencing (NGS) and the results were assayed by the changes in sgRNA representation after cell proliferation compared to the starting plasmid library. As a positive control, we performed parallel loss-of-function lethality screen using a constitutive sgRNA expression vector. As expected, the sgRNAs targeting a curated set of essential genes were strongly depleted following cell proliferation, whereas the representation of sgRNAs targeting nonessential genes was maintained (Fig. 4b). Similar results were obtained using DOX- and IPTG-inducible sgRNA expression vectors in the presence of their respective chemical inducers. To the contrary, we did not observe significant depletion of the sgRNAs against the essential or nonessential genes from the DOX- or IPTG-inducible sgRNA libraries after cell growth without addition of the inducers, validating their tightness in genome-scale screening (Fig. 4b). We selected four representative genes, including prohibitin 2 (*Phb2*), proteasome subunit alpha 6 (*Psma6*), ribosomal protein S14 (*Rps14*) and DNA polymerase epsilon (*Pole*), that are involved in distinct essential biological processes for cell survival. All the four independent sgRNAs targeting each gene were dramatically depleted from the constitutive, DOX- and IPTG-induced sgRNA expression libraries following cell proliferation, whereas their abundances were minimally affected in the non-induced conditions (Fig. 4c). For systematic gene-level analysis, we defined the lethality score of each gene to be the median log2 fold change in the abundance of all sgRNAs targeting the same gene comparing cells after proliferation to the plasmid DNA. While most gold standard essential genes were not modified using our inducible sgRNA expression vectors in the absence of chemical inducers, they were upon inducer addition and their lethality scores were well correlated with those in the constitutive screen (Fig. 4d). For statistical analysis, we used the MAGeCK (model-based analysis of genome-wide CRISPR-Cas9 knockout) algorithm [33] to score all the genes in our library for their lethality and obtained false discovery rate (FDR) for each gene. We then performed receiver operating characteristic (ROC) analysis using FDR as the classifier and relied on the reference sets of essential and nonessential genes to estimate true positive and false positive rates (Fig. 4e). We found that constitutive, DOX- and IPTG-induced sgRNA screens have very high performance in detection of essential genes, with the area under the curve (AUC) of the ROC curves > 0.80. To the contrary, screens in non-induced conditions using our inducible sgRNA vectors completely failed to distinguish essential and nonessential genes (AUC < 0.5). Collectively, these data demonstrate the tightness and efficiency of our conditional CRISPR-Cas9 genome editing systems in large-scale lethality screens.

### FACS-based pooled CRISPR screens using drug-inducible sgRNA expression libraries

**Fig. 5:**
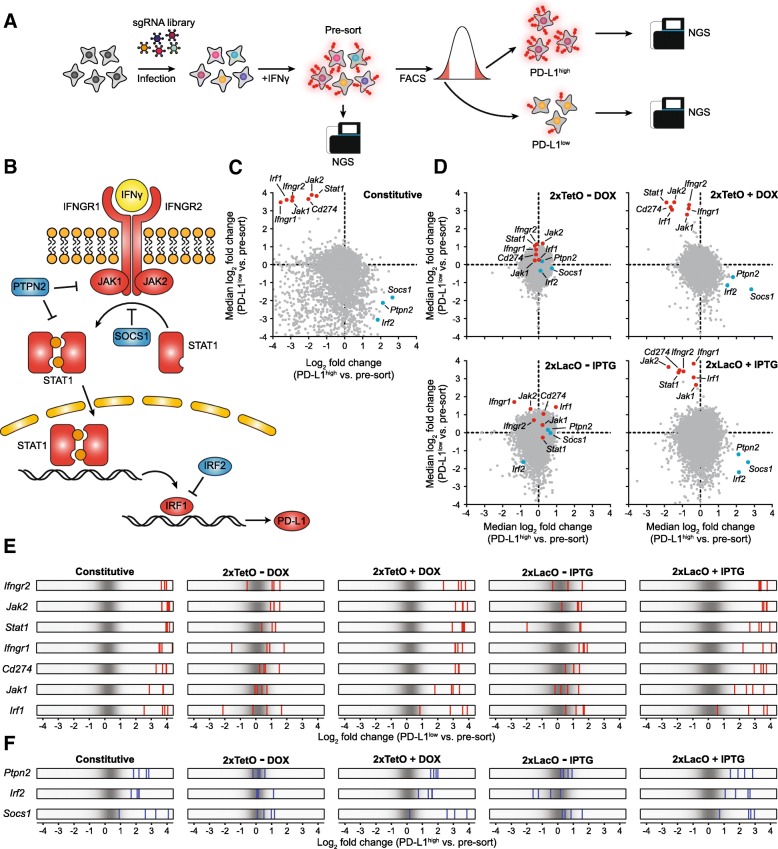
FACS-based pooled CRISPR screens using drug-inducible sgRNA expression constructs. (**a**) Schematic overview of FACS-based CRISPR screening approach to identify PD-L1 expression regulators. (**b**) Regulation of PD-L1 expression by IFNγ signaling pathway. Known positive PD-L1 regulators including PD-L1 itself are shown in red. Known negative PD-L1 regulators are shown in blue. (**c**) Scatter plot for the result of constitutive CRISPR screen. Each dot indicates median log2 fold change of all sgRNAs for one target gene. (**d**) Scatter plots for the screening results using DOX- or IPTG-inducible sgRNA vectors. Each dot indicates median log2 fold change of all sgRNAs for one target gene. 1 μg/mL DOX or 1 mM IPTG was used to induce the sgRNA expression. (**e**,**f**) Frequency histograms of the changes of sgRNA abundance in PD-L1^low^ (**e**) and PD-L1^high^ (**f**) cells versus pre-sort. sgRNAs targeting known PD-L1 positive regulating genes are shown by red and sgRNAs targeting known PD-L1 negative regulating genes are shown by blue

Expression of PD-L1 on tumor cells plays a critical role for cancer immune evasion [34, 35]. Treatment of interferon-γ (IFNγ) induced high level of PD-L1 expression on the surface of MC-38 cells (Additional file 1 :Figure S11) [36]. Surface PD-L1 expression can be efficiently interrogated using our drug-inducible sgRNA constructs with little background activity (Additional file 1 :Figure S12). Based on these observations, we set out to evaluate the performance of our drug-inducible CRISPR-Cas9 platforms in FACS-based genetic screens for PD-L1 modulators in IFNγ-treated MC-38 cells. Briefly, over 140 million MC-38 cells with stable Cas9 expression were transduced with the Brie genome-wide sgRNA library at a MOI of 0.3 in three biological replicates. After puromycin selection and IFNγ stimulation, we sorted cells based on high or low PD-L1 expression levels and then determined sgRNA abundance by NGS (Fig. 5a). We also amplified and sequenced sgRNAs from at least 40 million cells post IFNγ stimulation but prior to FACS (“pre-sort”) as a reference. We reasoned that the sgRNAs against positive regulators of PD-L1 expression would be enriched in PD-L1^low^ cells relative to pre-sort and the sgRNAs targeting negative regulators will be enriched in PD-L1^high^ cells relative to pre-sort. IFNγ-induced PD-L1 expression is primarily regulated by interferon regulatory factor 1 (IRF1) and IFNγ receptor signaling pathway, comprised of interferon gamma receptor 1 (IFNGR1), interferon gamma receptor 2 (IFNGR2), Janus kinase 1 (JAK1), Janus kinase 2 (JAK2), and signal transducer and activator of transcription 1 (STAT1) [37–39]. Conversely, protein tyrosine phosphatase non-receptor type 2 (PTPN2), suppressor of cytokine signaling 1 (SOCS1), and interferon regulatory factor 2 (IRF2) contribute to the attenuation of IFNγ signaling (Fig. 5b) [37, 40, 41]. In the screen using a constitutive sgRNA expression vector, we observed that the sgRNAs targeting all the key components of the IFNγ pathway and the PD-L1 gene itself (*Cd274*) were significantly enriched in PD-L1^low^ and concomitantly depleted in PD-L1^high^ cells, while the sgRNAs against *Ptpn2*, *Socs1* and *Irf2* were strongly enriched in PD-L1^high^ but dropped out of PD-L1^low^ cells (Fig. 5c and Additional file 2 :Table S2). These data demonstrate the validity of the screen setup. Next, we performed parallel FACS-based screens using our DOX- and IPTG-inducible sgRNA expression vectors in the presence or absence of their respective chemical inducers. Consistent with the results obtained in the constitutive screening, all the aforementioned positive and negative PD-L1 regulators were identified as strong hits in PD-L1^low^ and PD-L1^high^ cells, respectively, upon inducer addition (Fig. 5d-f and Additional file 1 :Figure S13&S14). In contrast, the fold-change distribution of sgRNAs targeting those genes was not significantly shifted in sorted cells relative to pre-sort control in non-induced cells (Fig. 5d-f and Additional file 1 :Figure S13&S14). We next used MAGeCK to rank all the genes in our library for enrichment in PD-L1^low^ or PD-L1^high^ cells relative to pre-sort. Using our inducible sgRNA expression vectors in the absence of chemical inducers, none of the genes in the Brie library were significantly enriched in PD-L1^low^ or PD-L1^high^ cells and the FDRs of the top screening hits were above 0.5 (Additional file 1 :Figure S15 and Table S3&S4). However, substantial number of genes, including the known positive and negative regulators of PD-L1, were impacted by the addition of inducers (Additional file 1 :Figure S15 and Table S3&S4). Taken together, our drug-inducible CRISPR-Cas9 systems enable robust control of genome interrogating activities in large-scale functional screening and represent a powerful tool to dissect causal gene function in dynamic and diverse biological processes.

## Discussion

High-throughput CRISPR-Cas9 screens using pooled sgRNA libraries provide an unbiased and precisely targeted method for the systematic assessment of gene function. In addition to catalog core and context-dependent fitness genes, CRISPR screens have been used to discover phenotypic modulators in a variety of biological processes, including drug resistance, protein expression, metabolism and viral infection. However, broader applications of this powerful technology in studying complex and multi-stage biological processes have been hampered by the constitutive activity of CRISPR-Cas9. For example, pluripotent stem cells and their differentiated progenies have been widely used for modeling human diseases, but it would be difficult to study gene function in terminally differentiated cells using constitutive CRISPR screens when the genes are also essential for the viability of progenitor cells. In such cases, the timing of genetic perturbation is critical and it is highly desirable that the activity of CRISPR-Cas9 can be regulated in an inducible manner so that the gene of interest can be functionally expressed prior to induction.

Previously, substantial effort has been devoted to the development of inducible CRISPR-Cas9 systems. However, most of those designs are not compatible with high-throughput genetic screening. For instance, induction by UV irradiation [14, 15], heat shock [23] or addition of cytotoxic chemicals such as rapamycin [19, 26], 4-hydroxytamoxifen [13, 16–19, 27] or BCL-xL inhibitors [28, 29] might cause undesirable biological effects and lead to confounding screening results. Photoactivable Cas9 approaches require specialized illumination devices which most laboratories are not equipped with [20, 42]. Placing the Cas9 gene under the control of tetracycline responsive element promoter has been shown to elicit significant background activity due to leaky Cas9 expression [10, 17, 43]. The split Cas9 architecture, in which the Cas9 protein is divided into two polypeptide chains and regulated by inducible dimerization, often results in suboptimal activity due to non-covalent protein dimerization [19–21, 26]. In other studies, fusion of Cas9 to large or repetitive sequences hampers its delivery by viral vectors [16, 17]. As a complementary approach to controlling Cas9 activity, Tang et al conjugated a blocking sequence and a theophylline-responsive aptazyme to sgRNA to control its conformation [25]. However, because the blocking sequence changes with its complementary sgRNA sequence, it is difficult to design and synthesize pooled sgRNA libraries and the blocking efficiency varies among different sgRNAs. Lac and Tet regulatory systems have been adopted to control sgRNA expression [12, 22, 44, 45]. However, little work has been performed to characterize their performances in large-scale CRISPR screens. In this study, we developed a robust drug-inducible CRISPR-Cas9 system that facilitates high-throughput gene manipulation.

In general, there are three key performance measures of an inducible CRISPR-Cas9 system: 1) the degree of background editing in the absence of inducer; 2) the degree of gene knockout upon inducer addition; 3) generality across different cell types. To find a satisfactory balance between leakiness and induction efficiency, we constructed multiple vectors with one or two copies of Tet or Lac operators inserted into the U6 promoters to achieve conditional sgRNA transcription. Next, we rigorously characterized our inducible sgRNA expression vectors in 12 cell lines from different species and tissues. In all the tested cell lines, we found that the U6 promoter variants containing two copies of TetO or LacO sequences showed little basal transcription in the non-induced state (Fig. 1e and Additional file 1 :Table S1). Importantly, off-state editing did not accumulate over time (Fig. 2a), demonstrating their utilities for long-term experiments. Upon addition of chemical inducers, we observed efficient gene knockout in a majority of the tested cell lines (activity score > 0.5, Fig. 1f and Additional file 1 :Table S1). However, the 2xTeO system exhibits inefficient DOX-induction in 4 T1 and 786–0, while the activity score of the 2xLacO system is < 0.5 in A-498 and 786–0. We did not see any correlation between induced sgRNA levels and gene editing rate (Additional file 1 Figure S5), suggesting there are other genetic or epigenetic factors contributing to the cell type specific activity. The precise molecular basis of this context dependency will need to be investigated in future studies; however, our system is viable for many commonly used cell lines. To demonstrate the efficiency of our drug-inducible sgRNA expression platforms in large-scale functional screens, we performed experimental side-by-side comparisons of constitutive and inducible CRISPR-Cas9 screens. In dropout screens for essential genes, these two approaches have similar performance in discriminating gold-standard essential and nonessential genes (Fig. 4). In FACS-based positive-selection screens, the two screening approaches showed similar sensitivity in detecting known PD-L1 expression regulators (Fig. 5). In the absence of chemical inducers, the non-induced CRISPR screens failed to identify any hit with statistical significance, suggesting minimal leakiness of our inducible platforms in large-scale settings.

Our DOX-inducible sgRNA expression system works with high efficiency in a hematopoietic reconstitution mouse model (Fig. 3), demonstrating the versatile use of our platform to generate temporal-specific gene knockout in hematopoietic stem cells and their differentiated progeny. We anticipate that pooled in vivo screens of gene function will be possible using this approach. Future investigations are needed to confirm the general applicability of our approach in a larger scale. Recently, in vivo CRISPR screens have been conducted using xenograft or syngeneic mouse models, enabling the dissection of causal gene functions in tumor growth, metastasis and tumor immune evasion [8, 37, 46–48]. In those studies, a pooled sgRNA library was first introduced to cells in culture. The resulting mutagenized cell library was then subcutaneously or intravenously transplanted into mice to assess phenotypes in vivo. While these studies are valuable for identifying cell-intrinsic properties of cancer cells, in vivo CRISPR screens using our experimental system will enable the study of gene function in the endogenous cells of hematopoietic origin, which are universally found in tumor microenvironment and play critical roles in tumor immune evasion. Future studies may also achieve spatial control over genome editing by expressing Cas9 from tissue-specific promoters.

In addition to its canonical use for targeted gene knockouts, CRISPR-Cas9 has been repurposed for a wide range of applications including transcription activation, transcription repression, genome labeling/imaging, histone modification, DNA methylation and base editing [49–51]. Since it is the sgRNA, not Cas9 under the control in our platform, our approach should be readily extensible to these applications without additional protein engineering effort. Moreover, our approach can be easily adapted to the emerging CRISPR-Cas13 systems for conditional RNA interference [52–54]. It is worth noting that the DOX- and IPTG-inducible systems can serve as an orthogonal pair, enabling independent control of the modulation of two genes.

## Conclusions

In conclusion, we have developed a drug-inducible CRISPR-Cas9 system that shows high cleavage efficiency upon induction but low background activity, and is applicable to a wide range of cell types both in vitro and in vivo. Most importantly, we demonstrate its utility and efficiency in genome-scale genetic screens. Our work expands the functional genomics toolbox and should enable the development of inducible CRISPR screens for understanding complex cellular and molecular mechanisms.

## Methods

### Cell culture

A-498 cells (ATCC, HTB-44) were cultured in Minimum Essential Medium Eagle (Sigma-Aldrich) plus 10% fetal bovine serum (FBS, ThermoFisher Scientific) and 100 U/mL penicillin/streptomycin (ThermoFisher Scientific). 786–0 (ATCC, CRL-1932), NCI-H1299 (ATCC, CRL-5803), CT26 (ATCC, CRL-2638) and 4 T1 (ATCC, CRL-2539) cells were cultured in RPMI Medium 1640 (ThermoFisher Scientific) plus 10% FBS, 1 mM sodium pyruvate (ThermoFisher Scientific), 0.45% D-(+)-Glucose (Sigma-Aldrich) and 100 U/mL penicillin/streptomycin. HEK293T (ATCC, CRL-3216) and LL/2 (ATCC, CRL-1642) cells were cultured in Dulbecco’s Modified Eagle Medium (ThermoFisher Scientific) plus 10% FBS and 100 U/mL penicillin/streptomycin.

### Generation of Cas9-stable cells

Cas9-stable cells were generated by infecting parental cell lines with a lentiviral construct expressing Cas9 and blasticidin-resistance gene in 12-well plates at 1000 xg for 2 h, in the presence of 8 μg/μL polybrene (Sigma-Aldrich). Plates were then returned to 37 °C with 5% CO_2_. Cells were incubated overnight and then selected by blasticidin (ThermoFisher Scientific).

### *EGFP* disruption assay

Parental cells and Cas9-expressing cells were infected with a lentivirus carrying an EGFP-2A-puroR cassette and an sgRNA targeting EGFP by spinfection in 12-well plates at 1000 xg for 2 h, in the presence of 8 μg/μL polybrene. Plates were then returned to 37 °C with 5% CO_2_. Cells were incubated overnight and then selected by puromycin (Sigma-Aldrich). Cells were passaged and cultured under puromycin selection for 3 days. The cells were then treated with various concentrations of doxycycline (Sigma-Aldrich) or IPTG (Sigma-Aldrich) for at least 5 days before flow cytometry analysis using an LSRFortessa X20 instrument (BD Biosciences).

### Expression of sgRNA by RT-qPCR

Total RNA was first extracted with RNeasy mini kit (QIAGEN) and treated with DNase I (QIAGEN) following the manufacturer’s instructions. The Transcriptor First Strand cDNA Synthesis Kit (Roche) was used for cfDNA synthesis. An sgRNA sequence-specific primer (5′-AAGCACCGACTCGGTGCCAC-3′) was added to the reaction mixture for reverse transcription for sgRNA detection. Quantitative real-time PCR was performed on a CFX384 Touch Real-Time PCR Detection System (Bio-Rad) with FastStart Essential DNA Green Master mix (Roche). Thermocycling parameters were defined as 95 °C for 10 min followed by 45 cycles of 95 °C for 10 s, 60 °C for 10 s and 72 °C for 10 s. The human or mouse gene encoding GAPDH is used as a reference to normalize the sgRNA expression level. The sequences of qPCR primers are shown below.

Forward primer for *EGFP*-specific sgRNA: 5′-GTGAACCGCATCGAGCTGAGTTT-3′.

Revers primer for *EGFP*-specific sgRNA: 5′- TTTCAAGTTGATAACGGACTAGCCT-3′.

Forward primer for murine *Gapdh*: 5′-CATGGCCTTCCGTGTTCCTA-3′.

Reverse primer for murine *Gapdh*: 5′- CCTGCTTCACCACCTTCTTGAT -3′.

Forward primer for human *GAPDH*: 5′- TCCAAAATCAAGTGGGGCGA-3′.

Reverse primer for human *GAPDH*: 5′- TGATGACCCTTTTGGCTCCC -3′.

### Genome-wide CRISPR lethality screens

> 140 million MC-38 cells were infected with the Brie sgRNA library at a multiplicity of infection (MOI) of 0.3 by spinfection in 12-well plates at 872 xg for 2 h, in the presence of 8 μg/μL polybrene (Sigma-Aldrich). Plates were then returned to 37 °C with 5% CO_2_. Cells were incubated overnight and then enzymatically detached using trypsin (ThermoFisher Scientifc). Cells for each of the three biological replicates were pooled and seeded into a 5-chamber CellSTACK (Corning) with 800 mL of fresh medium plus 6 μg/mL blasticidin and 3 μg/mL puromycin. After 3–4 days, cells were detached by trypsinization and counted. 40 million cells (~ 500-fold library coverage) were seeded into a new 5-chamber CellSTACK. After being passaged for > 10 populations doublings, for each replicate, 40 million cells were pelleted for genomic DNA extraction and > 100 million cells were used for FACS-based CRISPR screens. Genomic DNA was extracted using Quick-gDNA MidiPrep Kit (Zymo Research), according manufacturer’s instruction. The sgRNA sequences were amplified using the primers (listed below) harboring sequencing adaptors and barcodes. In order to achieve >500X coverage over the Brie library (assuming 5.8 μg of genomic DNA for 1 million cells), we performed 24 separate 100 μL PCR reactions with 10 μg genomic DNA in each reaction using ExTaq DNA Polymerase (Clontech) then combined the resultant amplicons. Samples were then purified with SPRIselect beads (Beckman Coulter) according to manufacturer’s instructions. Samples were quantified, mixed and sequenced on a NextSeq 500 (Illumina) by 75-bp single-end sequencing.

Forward primer mix for constitutive and IPTG-inducible sgRNA expression vector: 5′-AATGATACGGCGACCACCGAGATCTACACTCTTTCCCTACACGACGCTCTTCCGATCT(0–8 bp variable length sequence)TTGTGGAAAGGACGAAACACCG-3′.

Forward primer mix for DOX-inducible sgRNA expression vector: 5′-AATGATACGGCGACCACCGAGATCTACACTCTTTCCCTACACGACGCTCTTCCGATCT(0–8 bp variable length sequence) GATTATATATCTCCCTATCAGTGATAGACACCG-3′.

Reverse primer for constitutive, DOX- and IPTG-inducible sgRNA expression vector: 5′-CAAGCAGAAGACGGCATACGAGAT(8 bp barcode)GTGACTGGAGTTCAGACGTGTGCTCTTCCGATCTTCTACTATTCTTTCCCCTGCACTGT-3′.

### FACS-based CRISPR screens for PD-L1 regulators

> 40 million MC-38 cells that stably express Cas9 were infected with the Brie sgRNA library at MOI = 0.3 in three biological replicates. Following puromycin selection, the cells were treated with 20 ng/mL recombinant murine IFNγ (PeproTech) for 48 h. Prior to cell sorting, > 40 million cells were collected with trypsin as “pre-sort” for genomic DNA extraction. > 100 million cells were stained with Brilliant Violet 421-conjugated anti-mouse PD-L1 antibody (clone 10F.9G2, BioLegend) for 15 min on ice and washed with Cell Staining Buffer (BioLegend). The cells were then fixed with 1% paraformaldehyde (BioLegend) in Cell Staining Buffer for 30 min on ice and washed with Cell Staining Buffer. 5% PD-L1^low^ and 5% PD-L1^high^ cells were enriched by one round of FACS sorting using FACSAria Fusion cell sorter (BD Biosciences). At least two million PD-L1^low^ and PD-L1^high^ cells were collected for genomic DNA extraction using Quick-DNA FFPE Kit (Zymo Research). We performed 4 separate 100 μL PCR reactions with 2–6 μg genomic DNA in each reaction using ExTaq DNA Polymerase then combined the resultant amplicons. Samples were then purified with SPRIselect beads. Samples were quantified, mixed and sequenced on a NextSeq 500 by 75-bp single-end sequencing.

### Hematopoietic reconstitution with lentiviral infected LSK cells

All studies involving animals were performed according to protocols reviewed and approved by the Abbvie IACUC. C57/B6 Ly5.1 (Jackson order# 002014) and Cas9 knockin mice (Jackson order# 026197) were purchased from The Jackson Laboratory. The recipient mice were 6–8 week old (18-20 g) female Ly5.1 Pepboys. Donor mice were C57Bl/6’s age/sex matched. The procedures of generating bone marrow chimeras have been reported previously [30]. Briefly, LSK cells were isolated from the bone marrow of the Cas9 knockin mice using anti-CD117 microbeads (Miltenyi Biotec). Enriched cells were then cultured in StemSpan SFEM (STEMCELL Technologies) with recombinant stem cell factor, thrombopoietin, IL-7 and Flt3-ligand (PeproTech). Cell were then plated on RetroNectin (Clontech)-coated plates and spin-infected with lentivirus at 650 xg for 15 min at 30 °C. More than one day after infection, infected cells were washed in PBS and then injected intravenously into recipient mice that had been irradiated with two doses of 600 rads, 3 h apart. Three weeks after transplantation, the mice with their hematopoietic system reconstituted were randomly separated into two groups (five mice per group) and fed with regular or DOX-containing food (Harlan Teklad, TD.01306) for a week. Three additional weeks following DOX treatment, distinct immune cells from bone marrow, spleen and blood were subjected to flow cytometry analysis. The CD44 surface marker should decrease in the cells derived from the LSK donor if *CD44*-targeting sgRNA is induced. All animals were housed in specific pathogen free facilities and visually observed at least once daily for standard health checks. Any animals exhibiting poor body condition (ie. hunched posture, skin integrity loss) were euthanized. CO_2_ euthanasia was used to sacrifice the mice once the study was completed. The mice were placed into a clean euthanasia chamber with no gas. The gas was then turned on at a rate of 0.8 L/min to allow 10–30% of air displacement per minute. Once breathing had stopped, death was confirmed by cervical dislocation. The chamber was then opened to evacuate any remaining CO_2_ and cleaned for the next set of animals. Student’s t test was used for statistical analysis.

## Additional files

Additional file 1: **Figure S1.** Schematic of drug-inducible sgRNA expression lentiviral vectors. **Figure S2.** Nucleotide sequence presentation of the DOX- and IPTG-inducible U6 promoter variants used in this study. The TATA box is underlined. TetO sequences are highlighted in yellow. LacO sequences are highlighted in red. **Figure S3.** *EGFP* disruption activities using 1xTetO and 2xTetO constructs in response to DOX across different concentrations. Data represent mean ± SD (*n* = 3). **Figure S4.** *EGFP* disruption activities using 1xLetO and 2xLetO constructs in response to IPTG across different concentrations. Data represent mean ± SD (n = 3). **Figure S5.** Correlation of relative sgRNA expression and leakiness score (A) and activity score (B). Data represent mean ± SD (n = 3). **Figure S6.** Treatment of chemical inducers including DOX (A), IPTG (B), Shield-1 (C) and TMP (D) does not affect the cell viability of MC-38 and HEK293T cells. Data represent mean ± SD (n = 3). **Figure S7.** Treatment of chemical inducers does not affect EGFP fluorescence or Cas9 cleavage activity in MC-38 (A) or HEK293T (B) cells. Data represent mean ± SD (n = 3). **Figure S8.** High efficiency of hematopoietic reconstitution as indicated by the percentage of CD45.2-positive cells from different tissues. Data represent mean ± SD (*n* = 5). **Figure S9.** Composition of CD11b+, CD11c + and CD19+ cells from the spleen (A), bone marrow (B) and blood (C) of the hematopoietic-system-reconstituted mice. Data represent mean ± SD (n = 5). **Figure S10.** Treatment of DOX does not affect CD44 expression level in the hematopoietic-system-reconstituted mice. Data represent mean ± SD (n = 5). **Figure S11.** Surface PD-L1 expression in MC-38 and MC-38-*Cd274*^*−/−*^ cells with or without IFNγ (20 ng/mL) stimulation. **Figure S12.** Abolishment of surface PD-L1 expression using constitutive, DOX-inducible and IPTG-inducible sgRNA expression vectors in MC-38 cells. Data represent mean ± SD (n = 3). **Figure S13.** Scatter plots comparing the screening hits for positive PD-L1 regulators. (A) Correlation between induced and non-induced screening results using DOX-inducible sgRNA expression vector. Using median log2 fold change > 1 as the cutoff, 3 out of 31 screening hits were identified in the non-induced conditions, indicating 10% leakniess. (B) Correlation between DOX-induced and constitutive screen results. (C) Correlation between induced and non-induced screening results using IPTG-inducible sgRNA expression vector. Using median log2 fold change > 1 as the cutoff, 4 out of 31 screening hits were identified in the non-induced conditions, indicating 13% leakniess. (D) Correlation between IPTG-induced and constitutive screen results. **Figure S14.** Scatter plots comparing the screening hits for negative PD-L1 regulators. (A) Correlation between induced and non-induced screening results using DOX-inducible sgRNA expression vector. Using median log2 fold change > 1 as the cutoff, no hits were identified, representing minimal leakiness. (B) Correlation between DOX-induced and constitutive screen results. (C) Correlation between induced and non-induced screening results using IPTG-inducible sgRNA expression vector. Using median log2 fold change > 1 as the cutoff, no hits were identified, representing minimal leakiness. (D) Correlation between IPTG-induced and constitutive screen results. **Figure S15.** FDRs of the top 200 screen hits in FACS-based CRISPR screening for PD-L1 regulators. 1 μg/mL DOX or 1 mM IPTG was used to induce the sgRNA expression. **Table S1.** Leakiness scores and activity scores of the inducible systems in multiple cell lines. **Table S3.** False discovery rates (FDRs) and median log2 fold changes (FC) of the known PD-L1 positive regulating genes in the constitutive and inducible CRISPR screens. The calculation is based on the comparison of the sgRNA abundances in PD-L1^low^ versus pre-sort cells. **Table S4.** False discovery rates (FDRs) and median log2 fold changes (FC) of the known PD-L1 negative regulating genes in the constitutive and inducible CRISPR screens. The calculation is based on the comparison of the sgRNA abundances in PD-L1^high^ versus pre-sort cells. (DOCX 1013 kb)

Additional file 2: **Table S2.** Raw NGS count table for FACS-based CRISPR screening using constitutive sgRNA expression vector. (TXT 5224 kb)

## Abbreviations

AUC: area under the curve
CRISPR: clustered regularly interspaced short palindromic repeats
DHFR: dihydrofolate reductase
DOX: doxycycline
EGFP: enhanced green fluorescent protein
FACS: fluorescence-activated cell sorting
FDR: false discovery rate
IFNγ: interferon-γ
IPTG: isopropyl β-D-1-thiogalactopyranoside
LacI: lactose repressor
LacO: lactose operator
LSK: lineage−/Sca-1+/c-kit+
MAGeCK: model-based analysis of genome-wide CRISPR-Cas9 knockout
MOI: multiplicity of infection
NGS: next-generation sequencing
PD-L1: programmed death-ligand 1
ROC: performed receiver operating characteristic
RT-qPCR: quantitative reverse transcription PCR
TetO: tetracycline operator
TetR: tetracycline repressor
TMP: trimethoprim
vexGPF: violet-excited green fluorescent protein
