# Supplementary material for: Development of drug-inducible CRISPR-Cas9 systems for large-scale functional screening
Source: BMC Genomics. 2019 Mar 19;20:225. doi: 10.1186/s12864-019-5601-9 (PMC6425629; doi:10.1186/s12864-019-5601-9)
Supplement: Supplementary file 1 — Figure S1. Schematic of drug-inducible sgRNA expression lentiviral vectors. Figure S2. Nucleotide sequence presentation of the DOX- and IPTG-inducible U6 promoter variants used in this study. The TATA box is underlined. TetO sequences are highlighted in yellow. LacO sequences are highlighted in red. Figure S3. EGFP disruption activities using 1xTetO and 2xTetO constructs in response to DOX across different concentrations. Data represent mean ± SD (n = 3). Figure S4. EGFP disruption activities using 1xLetO and 2xLetO constructs in response to IPTG across different concentrations. Data represent mean ± SD (n = 3). Figure S5. Correlation of relative sgRNA expression and leakiness score (A) and activity score (B). Data represent mean ± SD (n = 3). Figure S6. Treatment of chemical inducers including DOX (A), IPTG (B), Shield-1 (C) and TMP (D) does not affect the cell viability of MC-38 and HEK293T cells. Data represent mean ± SD (n = 3). Figure S7. Treatment of chemical inducers does not affect EGFP fluorescence or Cas9 cleavage activity in MC-38 (A) or HEK293T (B) cells. Data represent mean ± SD (n = 3). Figure S8. High efficiency of hematopoietic reconstitution as indicated by the percentage of CD45.2-positive cells from different tissues. Data represent mean ± SD (n = 5). Figure S9. Composition of CD11b+, CD11c + and CD19+ cells from the spleen (A), bone marrow (B) and blood (C) of the hematopoietic-system-reconstituted mice. Data represent mean ± SD (n = 5). Figure S10. Treatment of DOX does not affect CD44 expression level in the hematopoietic-system-reconstituted mice. Data represent mean ± SD (n = 5). Figure S11. Surface PD-L1 expression in MC-38 and MC-38-Cd274−/− cells with or without IFNγ (20 ng/mL) stimulation. Figure S12. Abolishment of surface PD-L1 expression using constitutive, DOX-inducible and IPTG-inducible sgRNA expression vectors in MC-38 cells. Data represent mean ± SD (n = 3). Figure S13. Scatter plots comparing the screening hits for positive PD-L1 [file 12864_2019_5601_MOESM1_ESM.docx]

# Additional file 1. Supplementary figures and supplementary tables


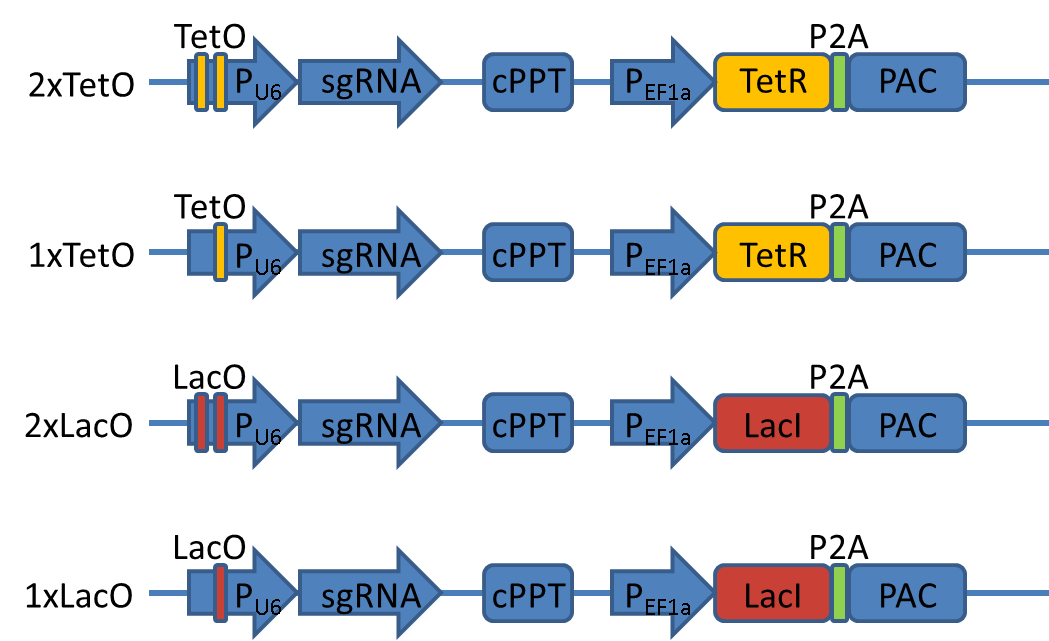


Figure S1. Schematic of drug-inducible sgRNA expression lentiviral vectors.

U6+2xTetO

GAGGGCCTATTTCCCATGATTCCTTCATATTTGCATATACGATACAAGGCTGTTAGAGAGATAATTAGAATTAATTTGACTGTAAACACAAAGATATTAGTACAAAATACGTGACGTAGAAAGTAATAATTTCTTGGGTAGTTTGCAGTTTTAAAATTATGTTTTAAAATGGACTATCATATGCTTACCGTAACTTGAAACTCCCTATCAGTGATAGAGATTATATATCTCCCTATCAGTGATAGACACC

U6+1xTetO

GAGGGCCTATTTCCCATGATTCCTTCATATTTGCATATACGATACAAGGCTGTTAGAGAGATAATTAGAATTAATTTGACTGTAAACACAAAGATATTAGTACAAAATACGTGACGTAGAAAGTAATAATTTCTTGGGTAGTTTGCAGTTTTAAAATTATGTTTTAAAATGGACTATCATATGCTTACCGTAACTTGAAACGTATTTCGATTTCTTGGCTTTATATATCTCCCTATCAGTGATAGACACC

U6+2xLacO

GAGGGCCTATTTCCCATGATTCCTTCATATTTGCATATACGATACAAGGCTGTTAGAGAGATAATTAGAATTAATTTGACTGTAAACACAAAGATATTAGTACAAAAAATTGTGAGCGGATAACAATTATTTCTTGGGTAGTTTGCAGTTTTAAAATTATGTTTTAAAATGGACTATCATATGCTTACCGTAACTTGAAAGTAATTGTGAGCGCTCACAATTATATATCTTGTGGAAAGGACGAAACACC

U6+1xLacO

GAGGGCCTATTTCCCATGATTCCTTCATATTTGCATATACGATACAAGGCTGTTAGAGAGATAATTAGAATTAATTTGACTGTAAACACAAAGATATTAGTACAAAATACGTGACGTAGAAAGTAATAATTTCTTGGGTAGTTTGCAGTTTTAAAATTATGTTTTAAAATGGACTATCATATGCTTACCGTAACTTGAAAGTAATTGTGAGCGCTCACAATTATATATCTTGTGGAAAGGACGAAACACC

Figure S2. Nucleotide sequence presentation of the DOX- and IPTG-inducible U6 promoter variants used in this study. The TATA box is underlined. TetO sequences are highlighted in yellow. LacO sequences are highlighted in red.


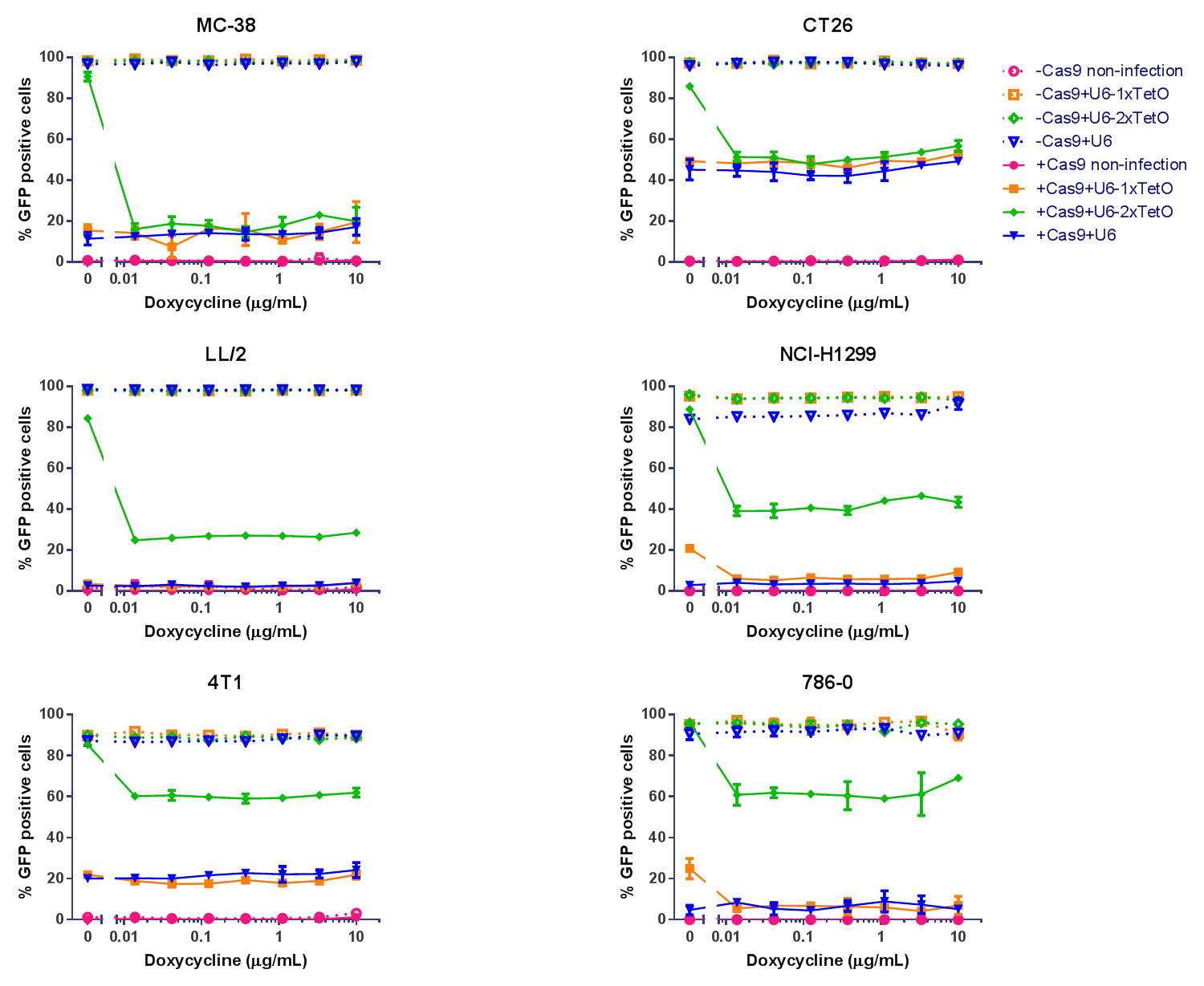


Figure S3. *EGFP* disruption activities using 1xTetO and 2xTetO constructs in response to DOX across different concentrations. Data represent mean ± SD (n = 3).


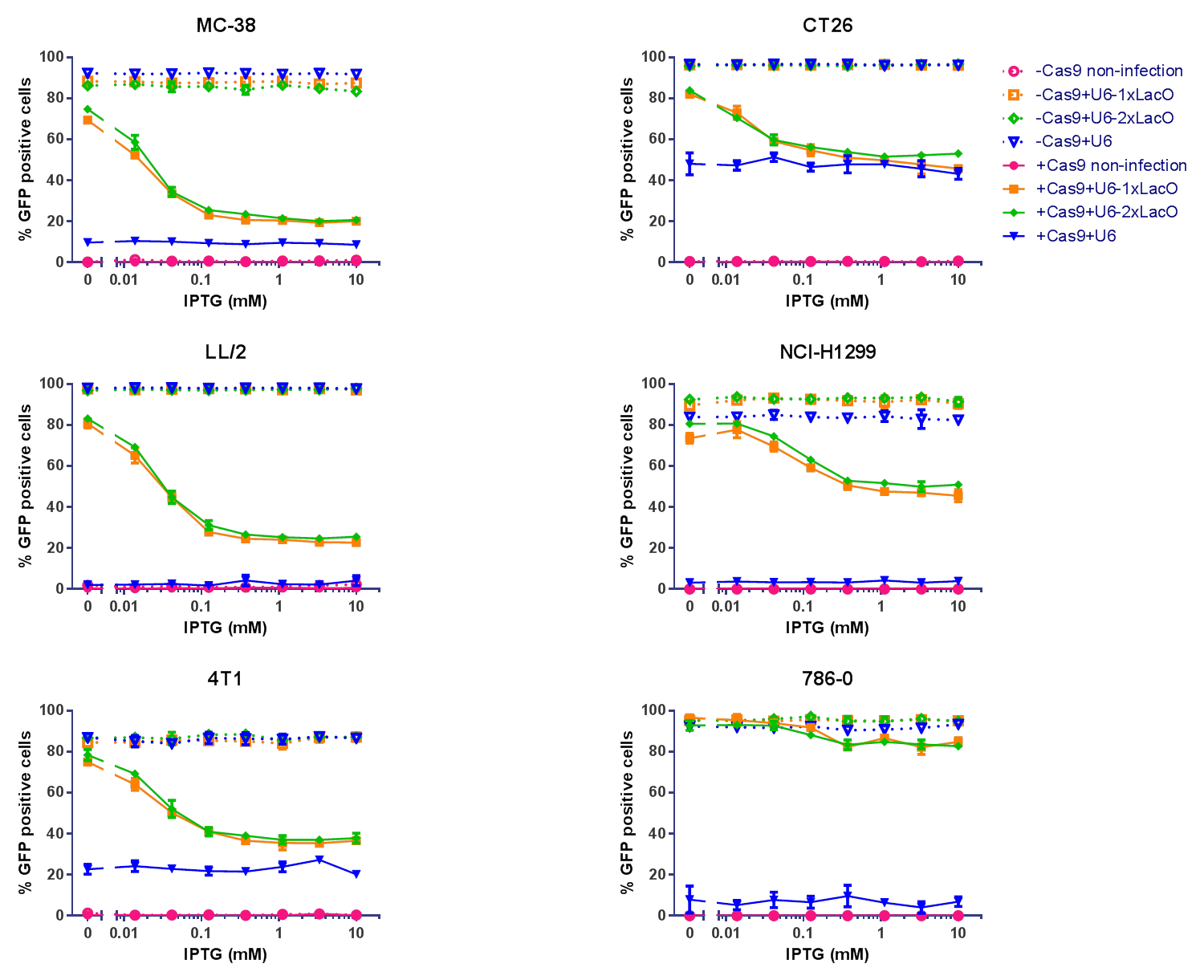


Figure S4. *EGFP* disruption activities using 1xLetO and 2xLetO constructs in response to IPTG across different concentrations. Data represent mean ± SD (n = 3).


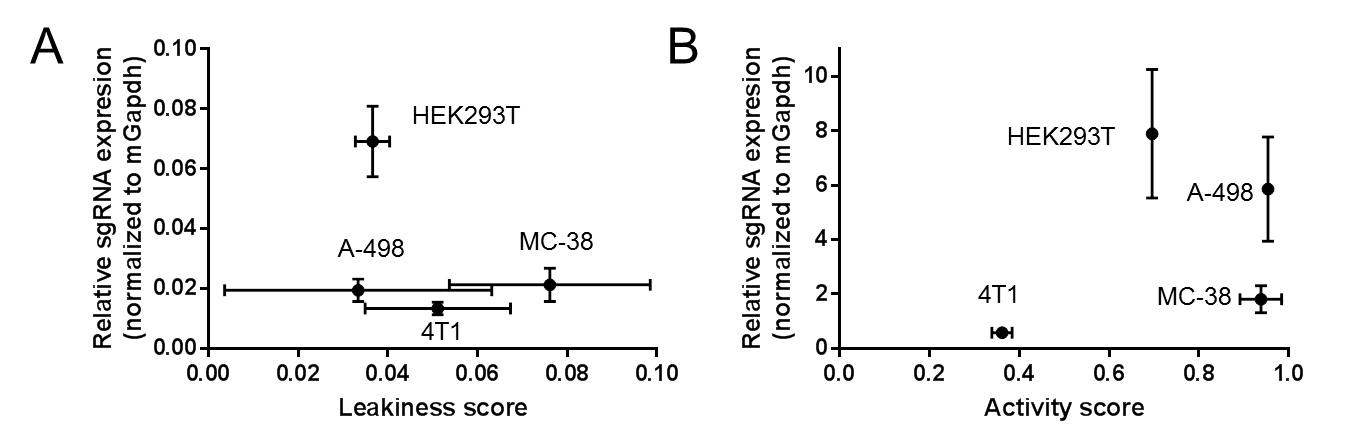


Figure S5. Correlation of relative sgRNA expression and leakiness score (A) and activity score (B). Data represent mean ± SD (n = 3).


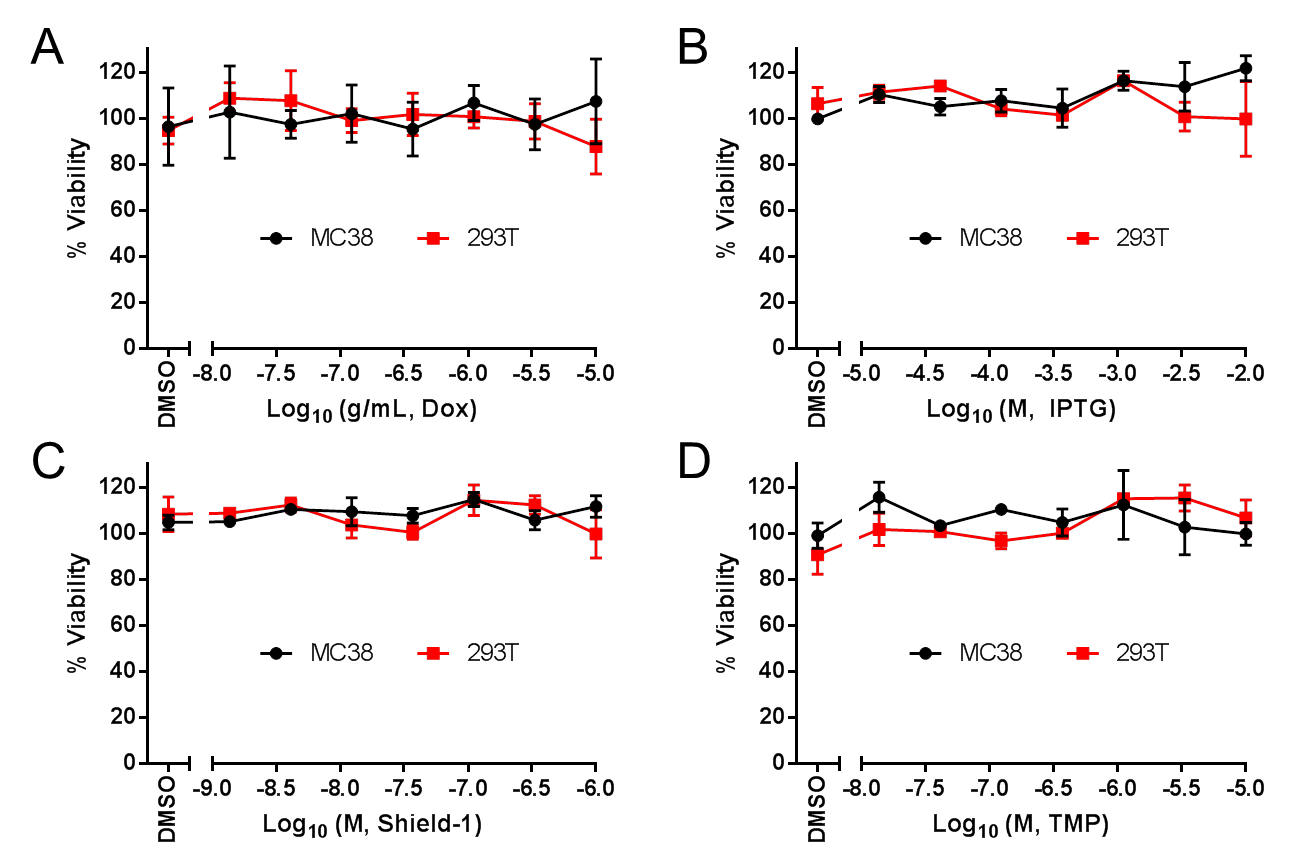


Figure S6. Treatment of chemical inducers including DOX (A), IPTG (B), Shield-1 (C) and TMP (D) does not affect the cell viability of MC-38 and HEK293T cells. Data represent mean ± SD (n = 3).


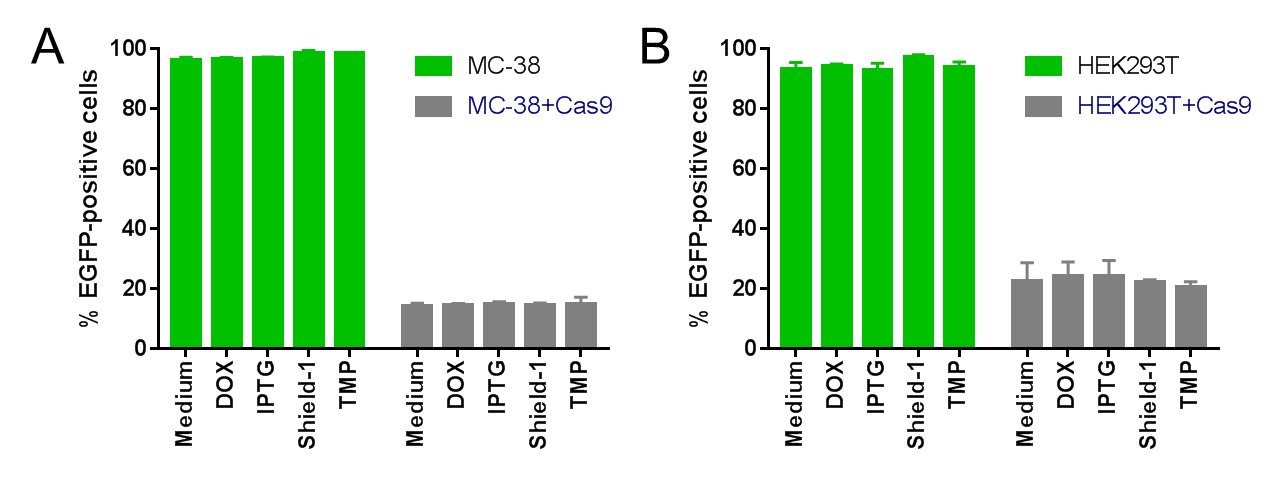


Figure S7. Treatment of chemical inducers does not affect EGFP fluorescence or Cas9 cleavage activity in MC-38 (A) or HEK293T (B) cells. Data represent mean ± SD (n = 3).


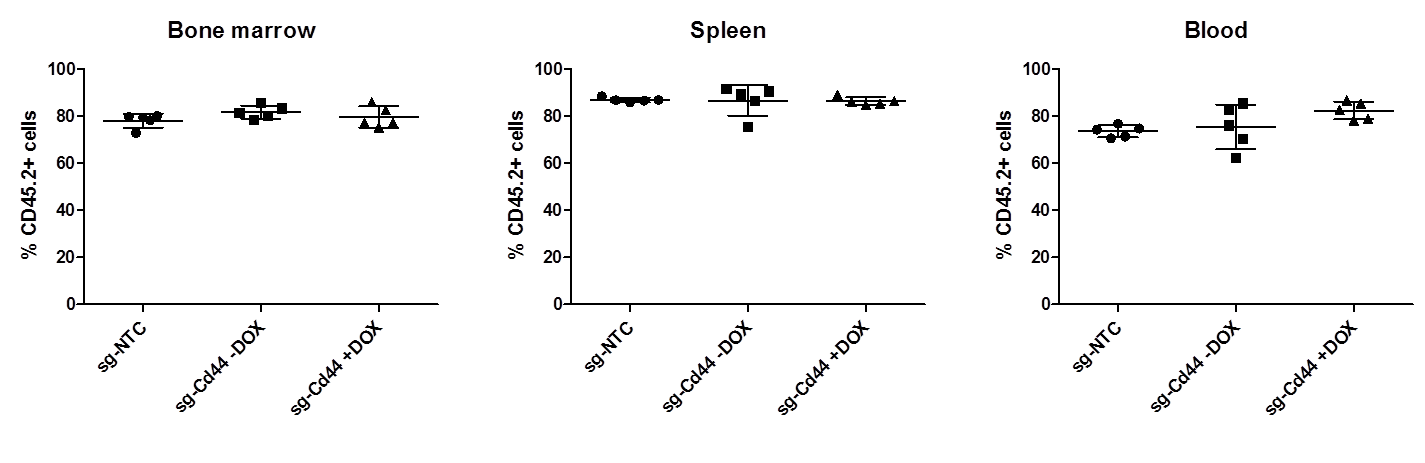


Figure S8. High efficiency of hematopoietic reconstitution as indicated by the percentage of CD45.2-positive cells from different tissues. Data represent mean ± SD (n = 5).


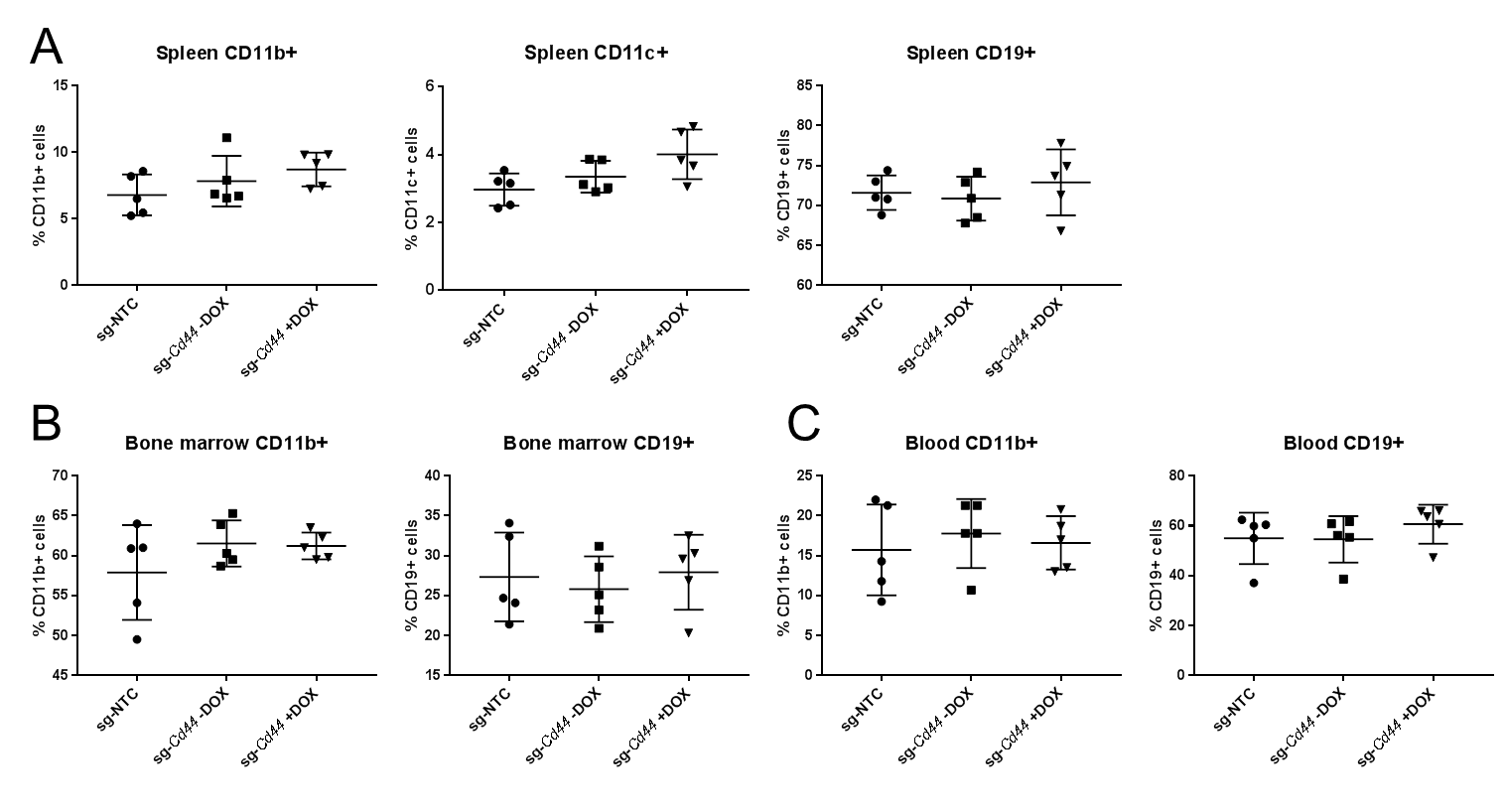


Figure S9. Composition of CD11b+, CD11c+ and CD19+ cells from the spleen (A), bone marrow (B) and blood (C) of the hematopoietic-system-reconstituted mice. Data represent mean ± SD (n = 5).


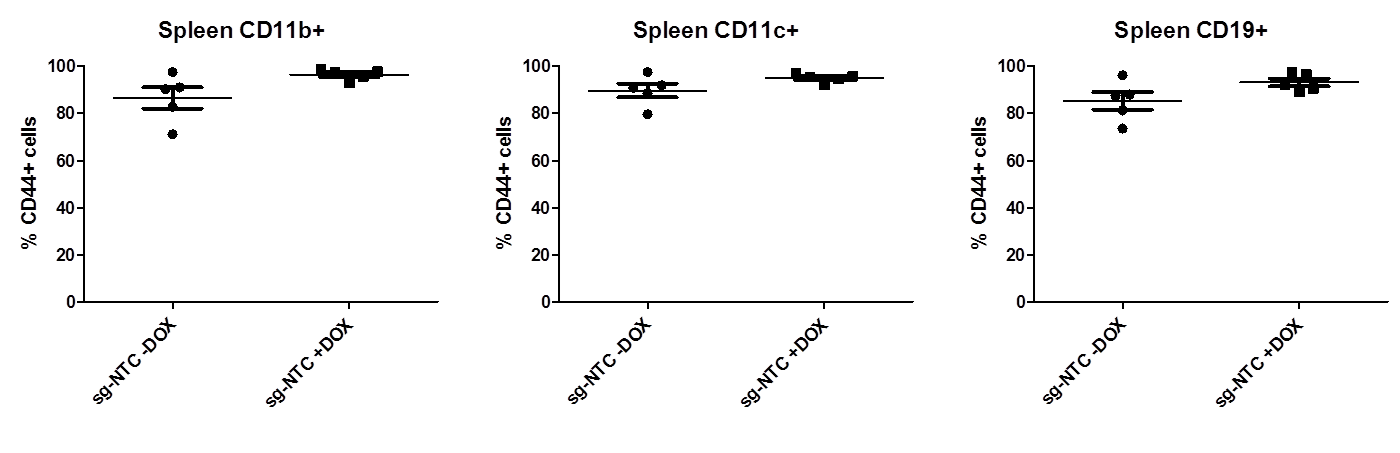


Figure S10. Treatment of DOX does not affect CD44 expression level in the hematopoietic-system-reconstituted mice. Data represent mean ± SD (n = 5).


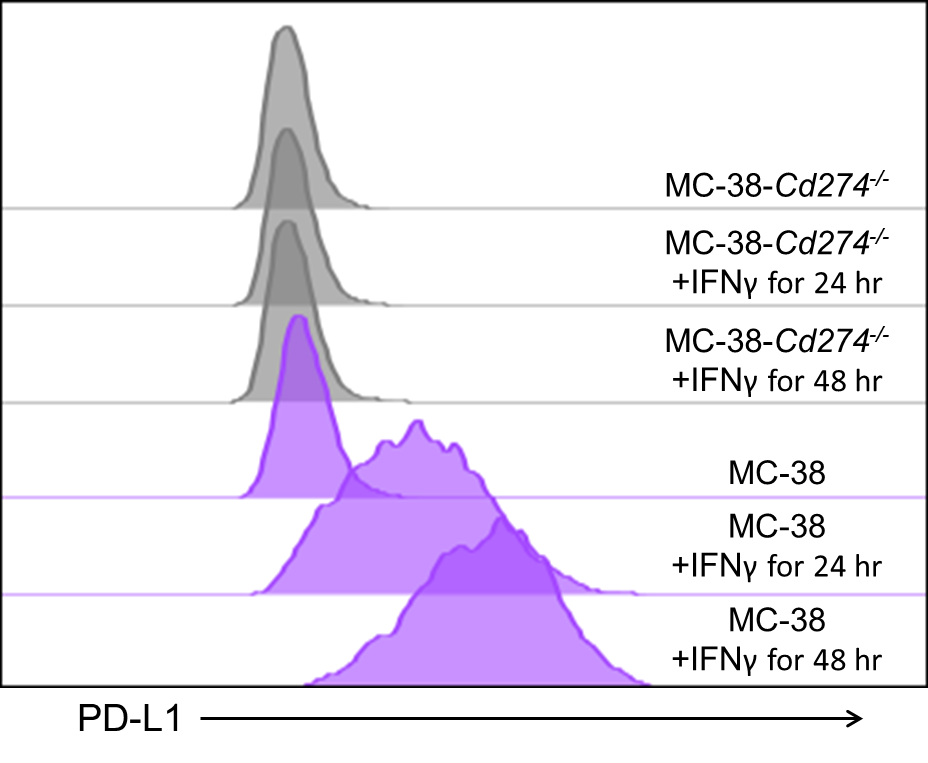


Figure S11. Surface PD-L1 expression in MC-38 and MC-38-*Cd274^-/-^* cells with or without IFNγ (20 ng/mL) stimulation.


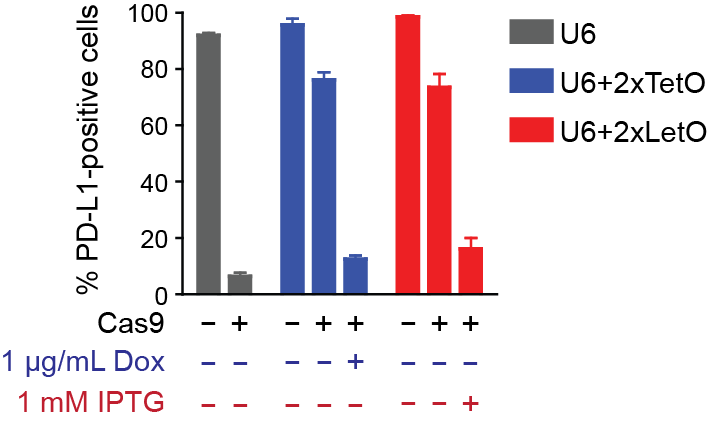


Figure S12. Abolishment of surface PD-L1 expression using constitutive, DOX-inducible and IPTG-inducible sgRNA expression vectors in MC-38 cells. Data represent mean ± SD (n = 3).


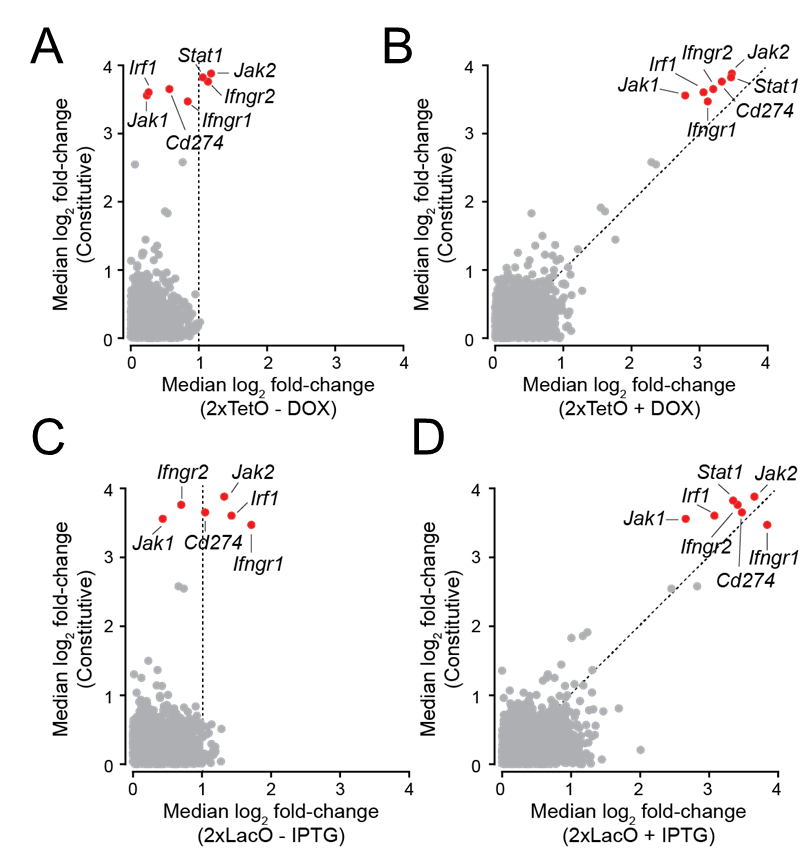


Figure S13. Scatter plots comparing the screening hits for positive PD-L1 regulators. (A) Correlation between induced and non-induced screening results using DOX-inducible sgRNA expression vector. Using median log2 fold change >1 as the cutoff, 3 out of 31 screening hits were identified in the non-induced conditions, indicating 10% leakniess. (B) Correlation between DOX-induced and constitutive screen results. (C) Correlation between induced and non-induced screening results using IPTG-inducible sgRNA expression vector. Using median log2 fold change >1 as the cutoff, 4 out of 31 screening hits were identified in the non-induced conditions, indicating 13 % leakniess. (D) Correlation between IPTG-induced and constitutive screen results.


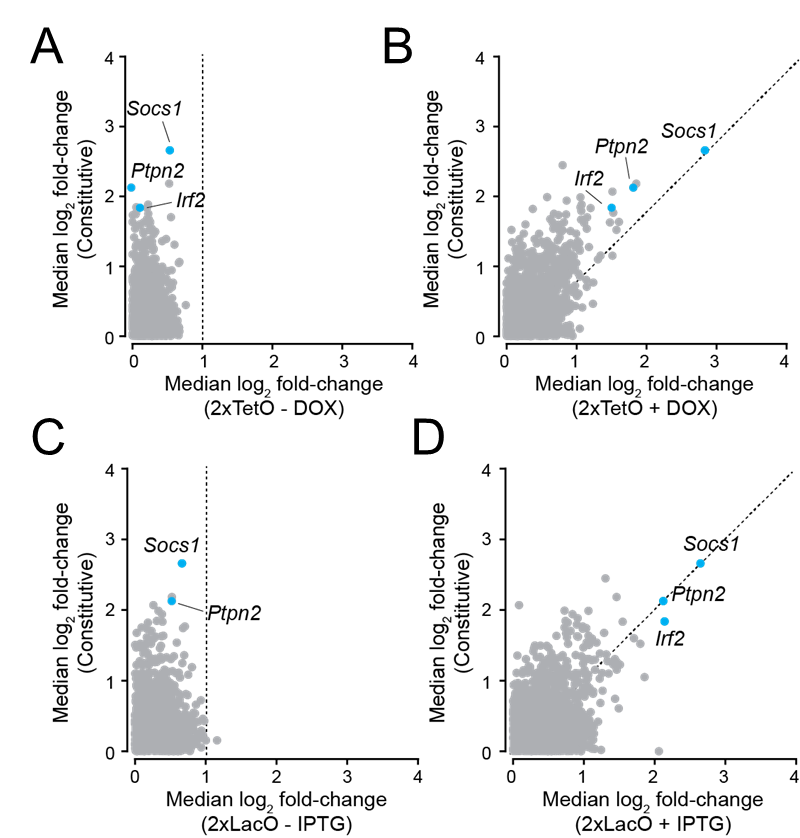


Figure S14. Scatter plots comparing the screening hits for negative PD-L1 regulators. (A) Correlation between induced and non-induced screening results using DOX-inducible sgRNA expression vector. Using median log2 fold change >1 as the cutoff, no hits were identified, representing minimal leakiness. (B) Correlation between DOX-induced and constitutive screen results. (C) Correlation between induced and non-induced screening results using IPTG-inducible sgRNA expression vector. Using median log2 fold change >1 as the cutoff, no hits were identified, representing minimal leakiness. (D) Correlation between IPTG-induced and constitutive screen results.


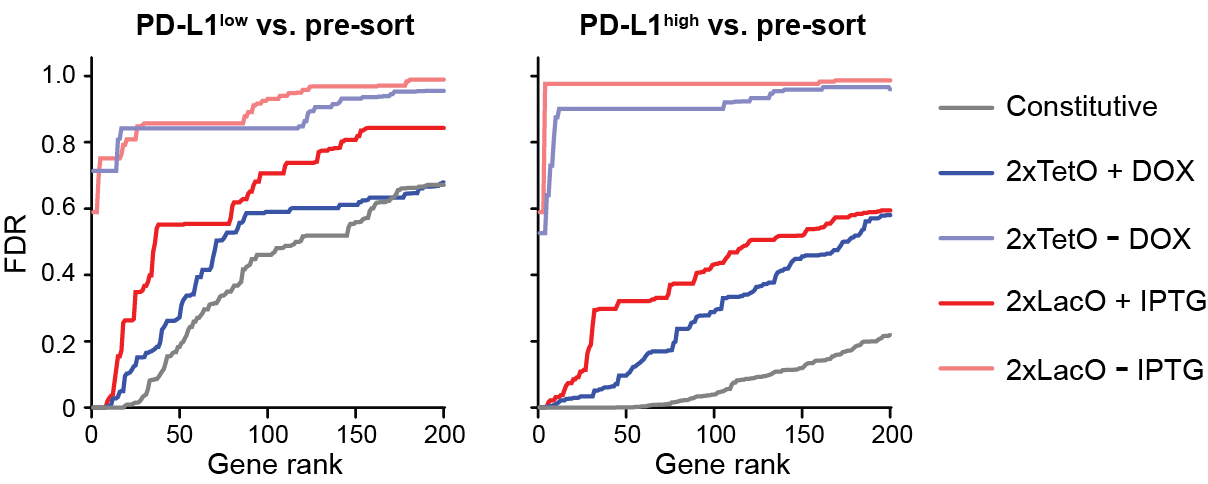


Figure S15. FDRs of the top 200 screen hits in FACS-based CRISPR screening for PD-L1 regulators. 1 μg/mL DOX or 1 mM IPTG was used to induce the sgRNA expression.

Table S1. Leakiness scores and activity scores of the inducible systems in multiple cell lines.

| **Cell line** | **Organism** | **Tissue** | **U6+1xTetO** | | **U6+2xTetO** | | **U6+1xLacO** | | **U6+2xLacO** | |
| --- | --- | --- | --- | --- | --- | --- | --- | --- | --- | --- |
|  |  |  | **Leakiness score** | **Activity score** | **Leakiness score** | **Activity score** | **Leakiness score** | **Activity score** | **Leakiness score** | **Activity score** |
| L-363 | Human | Blood | 0.51 | 1.00 | 0.09 | 0.99 | 0.10 | 0.51 | 0.17 | 0.77 |
| MC-38 | Mouse | Colon | 0.85 | 1.00 | 0.08 | 0.95 | 0.21 | 0.83 | 0.13 | 0.79 |
| A-498 | Human | Kidney | 0.73 | 1.00 | 0.03 | 0.95 | 0.06 | 0.53 | 0.00 | 0.41 |
| CT26 | Mouse | Colon | 0.50 | 0.93 | 0.12 | 0.89 | 0.15 | 0.97 | 0.13 | 0.93 |
| LL/2 | Mouse | Lung | 0.98 | 1.00 | 0.14 | 0.74 | 0.18 | 0.76 | 0.14 | 0.75 |
| HEK293T | Human | Kidney | 0.52 | 1.00 | 0.04 | 0.70 | 0.10 | 0.75 | 0.07 | 0.72 |
| LP-1 | Human | Blood | 0.73 | 1.00 | 0.00 | 0.68 | 0.12 | 0.72 | 0.11 | 0.65 |
| NCI-H1299 | Human | Lung | 0.78 | 1.00 | 0.07 | 0.60 | 0.18 | 0.55 | 0.13 | 0.52 |
| 4T1 | Mouse | Breast | 0.76 | 1.00 | 0.05 | 0.44 | 0.11 | 0.78 | 0.10 | 0.78 |
| 786-0 | Human | Kidney | 0.74 | 1.00 | 0.00 | 0.39 | 0.00 | 0.10 | 0.03 | 0.12 |
| OCI-LY10 | Human | Blood | N.T. | N.T. | 0.04 | 0.53 | N.T. | N.T. | 0.06 | 0.57 |
| THP1 | Human | Blood | N.T. | N.T. | 0.21 | 0.66 | N.T. | N.T. | 0.08 | 0.64 |

N.T.: not tested

Table S3. False discovery rates (FDRs) and median log2 fold changes (FC) of the known PD-L1 positive regulating genes in the constitutive and inducible CRISPR screens. The calculation is based on the comparison of the sgRNA abundances in PD-L1^low^ versus pre-sort cells.

| **Gene** | **Constitutive** | | **2xTetO -DOX** | | **2xTetO +DOX** | | **2xLacO -IPTG** | | **2xLacO +IPTG** | |
| --- | --- | --- | --- | --- | --- | --- | --- | --- | --- | --- |
|  | **FDR** | **Log2FC** | **FDR** | **Log2FC** | **FDR** | **Log2FC** | **FDR** | **Log2FC** | **FDR** | **Log2FC** |
| ***Ifngr1*** | 0.00029 | 3.47 | 0.81 | 0.84 | 0.00071 | 3.12 | 0.75 | 1.72 | 0.00062 | 3.84 |
| ***Ifngr2*** | 0.00029 | 3.76 | 0.71 | 1.14 | 0.00071 | 3.33 | 0.99 | 0.70 | 0.00062 | 3.41 |
| ***Jak1*** | 0.00029 | 3.56 | 0.99 | 0.24 | 0.00071 | 2.79 | 0.99 | 0.43 | 0.00062 | 2.66 |
| ***Jak2*** | 0.00029 | 3.88 | 0.84 | 1.18 | 0.00071 | 3.47 | 0.99 | 1.32 | 0.00062 | 3.66 |
| ***Stat1*** | 0.00029 | 3.83 | 0.99 | 1.06 | 0.00071 | 3.46 | 0.99 | -0.27 | 0.00062 | 3.35 |
| ***Irf1*** | 0.00029 | 3.61 | 0.99 | 0.26 | 0.00071 | 3.06 | 0.99 | 1.43 | 0.40 | 3.08 |
| ***Cd274*** | 0.00029 | 3.65 | 0.96 | 0.57 | 0.00071 | 3.20 | 0.99 | 1.05 | 0.00062 | 3.47 |

Table S4. False discovery rates (FDRs) and median log2 fold changes (FC) of the known PD-L1 negative regulating genes in the constitutive and inducible CRISPR screens. The calculation is based on the comparison of the sgRNA abundances in PD-L1^high^ versus pre-sort cells.

| **Gene** | **Constitutive** | | **2xTetO -DOX** | | **2xTetO +DOX** | | **2xLacO -IPTG** | | **2xLacO +IPTG** | |
| --- | --- | --- | --- | --- | --- | --- | --- | --- | --- | --- |
|  | **FDR** | **Log2FC** | **FDR** | **Log2FC** | **FDR** | **Log2FC** | **FDR** | **Log2FC** | **FDR** | **Log2FC** |
| ***Ptpn2*** | 0.00015 | 2.13 | 0.97 | 0.45 | 0.0012 | 1.81 | 0.99 | 0.52 | 0.0012 | 2.12 |
| ***Socs1*** | 0.00015 | 2.66 | 0.97 | 0.74 | 0.035 | 2.83 | 0.98 | 0.66 | 0.0012 | 2.65 |
| ***Irf2*** | 0.00015 | 1.84 | 0.53 | 0.11 | 0.028 | 1.50 | 1.00 | -0.85 | 0.0050 | 2.14 |
